# Menopause and vascular endothelial health: Is it all about the oestrogen?

**DOI:** 10.1113/EP092848

**Published:** 2026-05-30

**Authors:** Virginia R. Nuckols, Allyson I. Schwab, Lyndsey E. DuBose, Kerrie L. Moreau, Megan M. Wenner

**Affiliations:** ^1^ Department of Kinesiology and Applied Physiology University of Delaware Newark, DE USA; ^2^ Department of Health and Exercise Science Colorado State University Fort Collins, CO USA; ^3^ Division of Geriatric Medicine University of Colorado Anschutz Medical Campus Aurora, CO USA; ^4^ Geriatric Research, Education, and Clinical Center (GRECC) VA Eastern Colorado Health Care System Aurora, CO USA

**Keywords:** endothelium, perimenopause, reproductive hormones, women's health

## Abstract

Cardiovascular disease (CVD) is a leading cause of mortality in women, and CVD risk is accelerated during the menopause transition. This acceleration has traditionally been attributed to the hallmark decline in oestradiol with menopause. However, the menopause transition is also characterized by changes in other sex hormones that exert effects on the vascular endothelium including declines in progesterone and rising follicle stimulating hormone (FSH) and luteinizing hormone (LH) levels. The purpose of this review is to examine the relationships between sex hormones and vascular endothelial function in menopausal women. We emphasize data from clinical and translational studies investigating the effects of oestradiol, progesterone, FSH, LH, and testosterone on vascular endothelial function, and the putative underlying mechanisms including modulation of endothelin‐1 signalling, oxidative stress and inflammatory pathways.

## Abstract (highlights)

- **What is the topic of this review?**
  This review examines the associations between sex hormones and vascular endothelial function in menopausal women, with emphasis on hormones beyond oestradiol.
- **What advances does it highlight?**
  We highlight emerging evidence that follicle stimulating hormone, luteinizing hormone, progesterone and testosterone may contribute to vascular endothelial dysfunction during menopause. We explore underlying mechanisms by which sex hormones influence vascular endothelial function, such as endothelin‐1 signalling, oxidative stress and inflammation.

## 1 INTRODUCTION

The prevalence of cardiovascular disease (CVD) increases with age, but the rate of increase accelerates in midlife women, which corresponds with the timing of the menopause transition (El Khoudary et al., 2020). One proposed mechanism underlying the association between menopause and CVD is declines in endothelial function. Endothelial dysfunction is an early hallmark of vascular ageing and predicts future cardiovascular events (Rossi et al., 2004). Prior work indicated that endothelial function is preserved in women with ageing until the time of menopause, and thereafter the rate of decline in endothelial function is much greater in women compared to men (Celermajer et al., 1994). Indeed, numerous studies have demonstrated that postmenopausal women have lower endothelial function compared to young premenopausal women (Moreau et al., 2012, 2020). More recent studies have indicated that endothelial function starts to decline prior to menopause, among late premenopausal and perimenopausal women (Moreau et al., 2012; Wenner et al., 2024).

Traditionally, the accelerated development of CVD and reduced endothelial function in menopausal women has been attributed to changes in oestrogen (e.g., Celermajer et al. 1994). Indeed, oestrogen concentration is dramatically reduced in postmenopausal women compared to premenopausal women (Moreau et al., 2012; Wenner et al., 2024). Although preclinical models and smaller mechanistic human studies have demonstrated cardio‐protective effects of oestrogen, large scale randomized controlled trials investigating hormone therapy (HT) have yielded equivocal results regarding cardiovascular harm and benefit (Schwab et al., 2025). As such, the influence of sex hormones, including and beyond oestrogen, on changes in cardiovascular function with advancing reproductive age remains unclear. In this review, we provide a brief summary of the actions of sex hormones on the cardiovascular system, in particular the vascular endothelium, and highlight recent data investigating hormonal mechanisms contributing to declines in endothelial function with natural menopause.

## 2 CHANGES IN SEX HORMONES WITH AGEING AND MENOPAUSE

Sex hormone production in women is regulated through the hypothalamic–pituitary–ovarian axis. Release of luteinizing hormone (LH) and follicle stimulating hormone (FSH) by the anterior pituitary stimulate ovarian production of oestrogen and progesterone. The present review is focused on 17β‐oestradiol. Follicles within the ovary synthetize and secrete oestrogen and inhibin, which suppresses FSH and stimulates a LH ‘surge’ that initiates the process of ovulation. Following ovulation, LH stimulates the corpus luteum formed from the ruptured follicular cells to produce and secrete progesterone, which in turn modulates endometrial maturation (Herbison, 2016).

**FIGURE 1:**
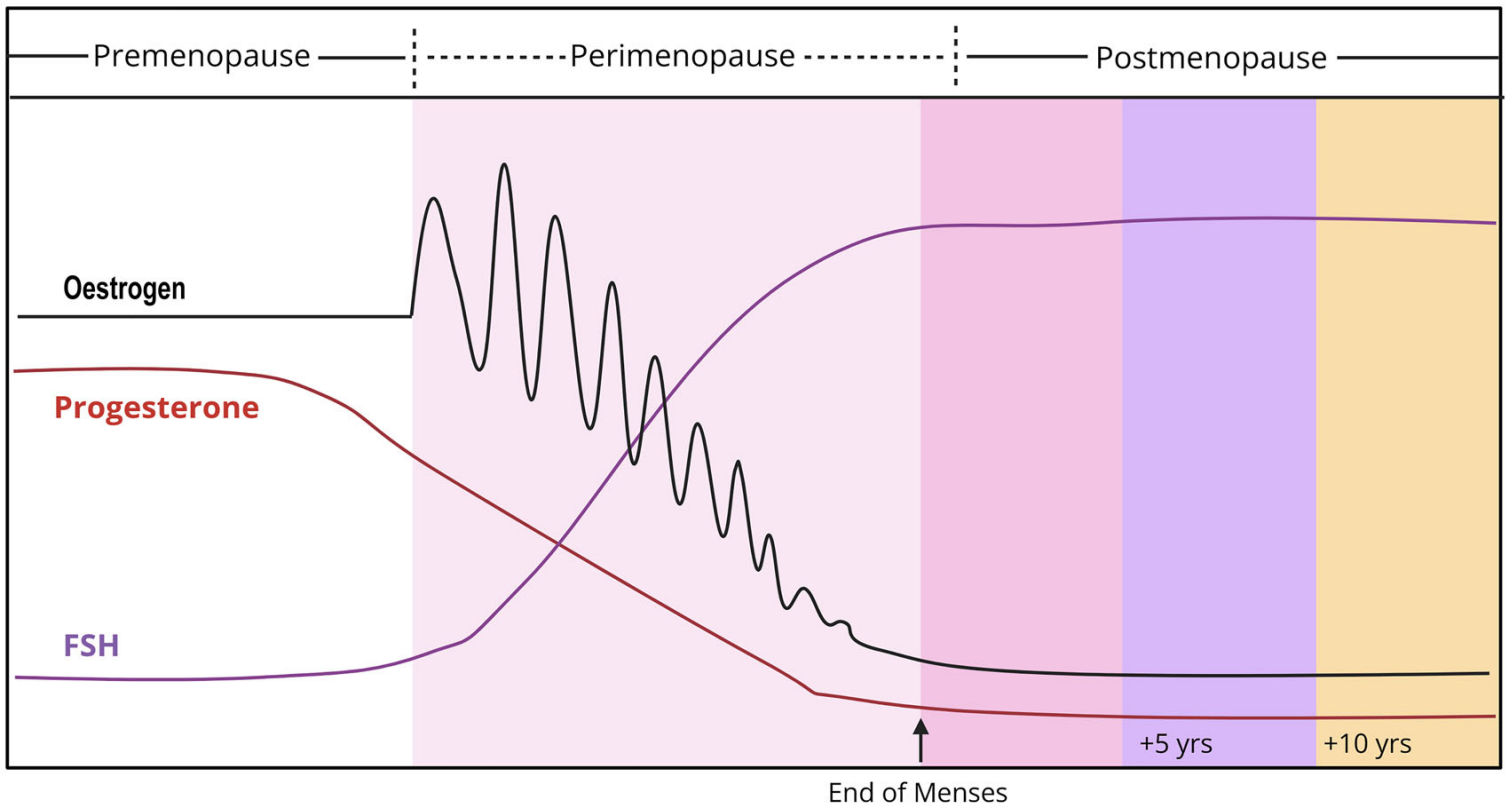
Hormone fluctuations in women across the menopause transition. Created in BioRender, adapted from Davis et al. (2015).

The menopause transition is marked by changes in the regularity and duration of menstrual cycles, due to both diminishing ovarian reserves and altered central sensitivity to oestradiol that disrupts feedback mechanisms on the hypothalamic–pituitary–ovarian axis (Weiss et al., 2004). Oestradiol fluctuates significantly as ovarian function declines (i.e., perimenopause), associated with aberrant menstrual cycle durations and menopausal symptoms. In general, longitudinal data indicate that circulating oestradiol and progesterone decline to consistently low levels and FSH increases in the years before the final menstrual period (Randolph et al., 2011), as represented in Figure 1. The onset of natural menopause is defined by 12 consecutive months without menses, at an average age of 50–51 years (Zhu et al., 2018). It is important to note that the longitudinal patterns of change in oestradiol and FSH across the menopause transition are not uniform; rather, distinct trajectories have emerged delineated by variable patterns and rates of change in oestradiol and FSH preceding and after the final menstrual period (Tepper et al., 2012). Cross‐sectional data with change‐point analyses in women aged 18–70 years indicate that age‐related reductions in oestradiol and progesterone begin at approximately 44 and 48 years of age, respectively (Wenner et al., 2024). Age‐related increases in gonadotropins FSH and LH begin even earlier in the lifespan at ∼33 years of age, well before oestradiol and progesterone. Despite this, the mechanistic role of gonadotropins in the progression of CVD risk in women has not been widely investigated.

Testosterone generally declines with chronological age in women, not concomitant with the menopause transition (Burger et al., 2000). The bioavailability of testosterone is modulated by sex hormone binding globulin (SHBG) (Selby, 1990), which declines modestly across the menopause transition resulting in a higher free androgen index (testosterone:SHBG) (Burger et al., 2000). Androstenedione, a precursor to testosterone, is similarly lower across the menopause transition (Wang et al., 2025).

## 3 ACTIONS OF SEX HORMONES ON THE CARDIOVASCULAR SYSTEM

### 3.1 Overview of menopausal hormone therapy clinical trials on cardiovascular endpoints

Several seminal clinical trials have evaluated the safety and efficacy of HT administered to ‘replace’ the loss of oestradiol, for primary CVD prevention in menopausal women. For a comprehensive review, the reader is referred to Schwab et al. (2025). Briefly, in 2002, the Women's Health Initiative (WHI), a randomized controlled trial, was discontinued early and reported findings suggesting that HT increased CVD risk in postmenopausal women (Rossouw et al., 2002). Further analysis of the WHI data revealed that the CVD risk associated with HT was largely driven by older postmenopausal women, given that HT initiated earlier after the onset of menopause (<10 years) was associated with reduced CVD risk and total mortality (Hsia et al., 2006). This ‘timing hypothesis’ was prospectively tested in the Kronos Early Estrogen Prevention Study (KEEPS) trial. HT was initiated within 3 years of the final menstrual period as either low‐dose oral conjugated equine oestrogen or transdermal oestrogen with oral progesterone (Taylor et al., 2017). HT was beneficial for vasomotor symptoms, but there appeared to be no cardiovascular effect measured by carotid artery intima–media thickness. The Early versus Late Intervention Trial with Estradiol (ELITE) trial similarly found that progression of atherosclerosis was attenuated in the HT treatment group compared with placebo among the early postmenopausal women (<6 years since menopause) but not late postmenopausal women (≥10 years) (Hodis et al., 2016). Importantly, differences in health status, menopausal age and onset, and HT administration all contribute to the observed differences in these landmark trials. Accordingly, the 2022 Menopause Society position statement notes that the extant evidence does not support HT as a primary prevention strategy for CVD (Faubion et al., 2022).

### 3.2 Influence of sex hormones in vascular endothelial function

The vascular endothelium is a single layer of cells lining the blood vessels and releases vasoactive substances to maintain homeostasis and modulate vascular tone. Endothelial cells synthesize signalling molecules such as nitric oxide (NO) and endothelin (ET‐1; see ‘Integrative mechanisms’). NO is synthesized by endothelial nitric oxide synthase (eNOS) and diffuses into the adjacent vascular smooth muscle cells to elicit smooth muscle relaxation and vasodilation (Moncada & Higgs, 2006). Accordingly, the vascular endothelium plays a critical role in maintenance of healthy vascular function. Flow‐mediated dilation (FMD) is a common, non‐invasive measure of conduit artery (i.e., macrovascular) endothelial function in humans, reflecting the vasodilatory response to increased mechanical shear stimulus that is predominantly NO‐mediated (Thijssen et al., 2011). Lower FMD is a hallmark of CVD risk, and predicts the development of hypertension in healthy early postmenopausal women (Rossi et al., 2004).

Microvascular endothelial function is assessed by administration of vasoactive pharmacological agents (e.g., acetylcholine) or local heating and quantified by blood flow response (Holowatz et al., 2008). Microvascular endothelial dysfunction is thought to reflect early vascular pathophysiological processes that may precede and contribute to downstream conduit artery dysfunction (Gates et al., 2009; Lockhart et al., 2009). Cutaneous microvascular endothelial function reflects microcirculatory function in end‐organ vascular beds and is impaired in hypertension as well as chronological and reproductive ageing, like FMD (Holowatz et al., 2008; Wenner et al., 2017).

To date, the majority of studies have focused on the role of oestradiol in the menopause‐related decline in endothelial function. However, other sex hormones are altered with ageing and menopause and may potentiate or counter the effect of oestradiol or exert independent effects on the vasculature.

#### 3.2.1 Oestradiol

**FIGURE 2:**
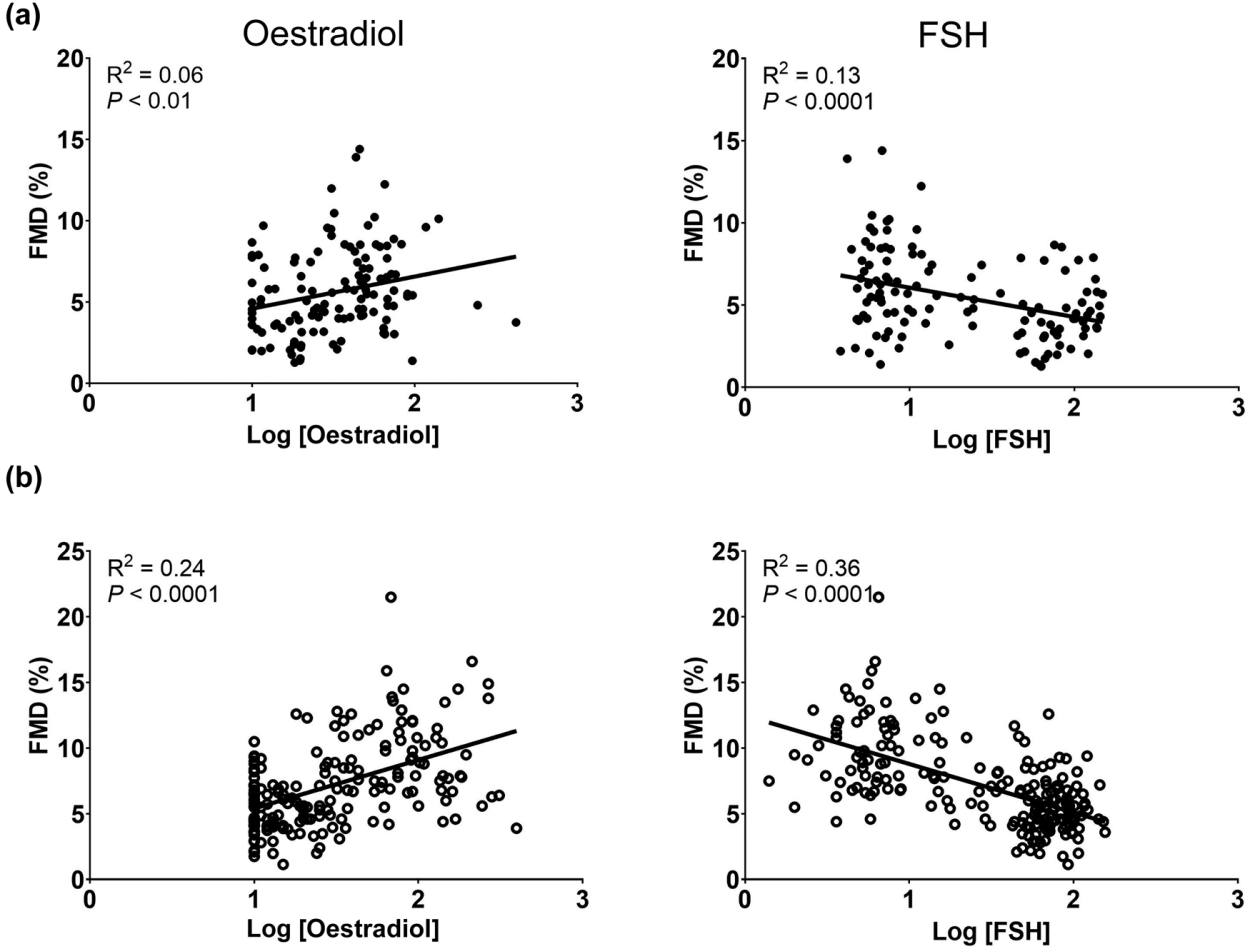
Cross‐sectional associations of oestradiol and follicle stimulating hormone (FSH) with vascular endothelial function in women across the lifespan. Flow mediated dilation (FMD) was measured in two separate cohorts of pre‐, peri‐ and postmenopausal women aged 18–70. The associations between FSH and FMD were consistently greater than those of oestradiol and FMD. (a) University of Delaware (*n* = 121) (Wenner et al., 2024) and (b) University of Colorado at Anschutz (*n* = 193) (Moreau et al., 2012).

**FIGURE 3:**
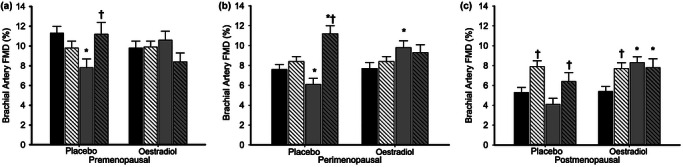
Acute antioxidant treatment improves endothelial function in oestrogen‐deficient women. Flow‐mediated dilation (FMD) was measured in (a) pre‐, (b) peri‐, and (c) postmenopausal women during saline (filled bars) and vitamin C infusion (hatched bars) at baseline (black bars) and under endogenous ovarian hormone suppression via GnRH antagonist (grey bars) plus placebo or exogenous oestradiol treatment. Data are means ± SE. *P < 0.01 vs. baseline saline of the same group; †*P* < 0.05 vs. GnRH antagonist plus add‐back saline of the same group. Reproduced with permission (Moreau et al., 2020).

FMD is progressively lower across stages of menopause transition in cross‐sectional analyses, concomitant with reductions in ovarian function (Moreau et al., 2012, 2020). Further, FMD is lower in women who underwent menopause at the average age of onset (∼50 years) compared with later onset (∼57 years) (Darvish et al., 2025). In cross‐sectional analyses, endogenous oestradiol is positively associated with FMD in women over the lifespan (Figure 2). In postmenopausal women specifically, oestradiol is correlated with FMD independently of chronological age, but this association is attenuated with adjustment for traditional CVD risk factors (e.g., blood pressure, body mass index) and time since menopause (Mathews et al., 2019). This finding is aligned with the evidence that the effect of menopause‐related declines in oestradiol on endothelial dysfunction is mediated in part through indirect pathways, such as adverse cardiometabolic risk profile (Clegg et al., 2017). However, intervention studies provide support for the independent role of oestradiol in endothelial function. Moreau et al. (2020) demonstrated that short‐term ovarian hormone suppression via gonadotropin‐releasing hormone antagonist (GnRH_ant_) elicited a decrease in FMD among premenopausal and, to a lesser extent, perimenopausal women but not postmenopausal women (Figure 3) suggesting that the contribution of endogenous ovarian hormones to endothelial function is attenuated across the menopausal transition. However, exogenous oestradiol administration rescued the GnRH_ant_‐induced impairment in FMD in pre‐ and perimenopausal women, and increased FMD in postmenopausal women (Moreau et al., 2020). This finding is consistent with meta‐analytic results showing chronic HT improves FMD in postmenopausal women (Gu et al., 2024). These findings collectively provide strong evidence that oestradiol, and loss thereof with declining ovarian function, is a causal factor in impaired endothelial function with the menopause transition.

The menopause‐related decline in oestradiol likely contributes to endothelial dysfunction in part via reduced eNOS activation and consequently reduced NO production. The effects of oestradiol are mediated largely through receptor subtypes ERα and ERβ.  More recently, a G protein‐coupled oestrogen receptor (GPER) subtype was identified and implicated in oestradiol‐mediated endothelial regulation (Prossnitz & Barton, 2023). ERα, ERβ and GPER subtypes are expressed on endothelial cells, though distinct roles remain to be fully elucidated (Mendelsohn & Karas, 1999). ERs modulate endothelial function via transcriptional upregulation of eNOS expression (MacRitchie et al., 1997) as well as rapid, non‐transcriptional mechanisms via membrane receptors (Russell et al., 2000). Oestradiol induces rapid eNOS activation and production of NO via ERα and GPER subtypes (Fredette et al., 2018; Gavin et al., 2009). Venous endothelial cell expression of ERα is lower in postmenopausal compared with premenopausal women (Gavin et al., 2009). Thus, the decline in endothelial function associated with menopause may be attributable to both reductions in oestradiol and oestrogen receptor expression. For detailed description of these signal transduction mechanisms, see Chambliss & Shaul (2002).

#### 3.2.2 Progesterone

Like oestradiol, the inflection point at which progesterone begins to exhibit age‐related decline occurs at age ∼48 (Wenner et al., 2024). Additionally, synthetic progestins are often used in combination with oestradiol‐based HT to mitigate endometrial dysregulation after menopause. As such, the effects of oestradiol and progesterone on vascular endothelial function in postmenopausal women are challenging to delineate. Among premenopausal women undergoing a controlled hormone intervention (i.e., endogenous hormone suppression), transdermal oestradiol improved FMD and this effect was abolished when oral micronized progesterone was added (Miner et al., 2011). However, oral progesterone alone did not blunt FMD in premenopausal women. Similarly, progestin (medroxyprogesterone acetate, MPA) administration reversed the favourable effect of oral oestrogen HT on FMD in postmenopausal women in a dose‐dependent manner (Wakatsuki et al., 2001). In contrast, the addition of micronized progesterone to transdermal oestrogen HT did not antagonize the improvement in FMD observed in postmenopausal women (Gerhard et al., 1998). Micronized progesterone had no adverse effect on endothelial function when administered alone in postmenopausal women (Prior et al., 2014). Acute infusion of progesterone also did not alter endothelium‐dependent vasodilation in postmenopausal women (Mather et al., 2000). These equivocal findings are likely due to differing chemical structures of progesterone versus synthetic progestins that have divergent effects on the vasculature. Indeed, progesterone increases NO production in vitro in human endothelial cells, whereas MPA has no effect (Simoncini et al., 2004). Further investigation is needed to delineate the impact of endogenous and various synthetic progesterone on endothelial function across the menopause transition.

#### 3.2.3 FSH and LH

Few studies have evaluated the independent roles of FSH or LH in endothelial function. FSH and LH are inversely associated with FMD across the adult lifespan in women in two separate cohorts (Figure 2; Moreau et al., 2012; Wenner et al., 2024) and among a sample of women at midlife (Serviente & Witkowski, 2019). Moreover, the association between advancing age and lower FMD was mediated in part by FSH level, but not oestradiol (Wenner et al., 2024). This finding is consistent with the idea that rising FSH due to chronological and/or reproductive ageing may contribute in part to the observed impairments in endothelial function with menopause. However, the mechanisms that underlie this association are largely unknown. FSH and LH stimulate atherogenesis via phosphoinositide 3‐kinase (PI3K)/Akt signalling pathways in human endothelial cells (Meng et al., 2019), and the increase in FSH with menopause is directly associated with subclinical atherosclerotic progression (carotid intima–media thickness) in humans (El Khoudary et al., 2016). These convergent findings indicate that gonadotropins may be proatherogenic, which is precipitated by impaired endothelial function (Davignon & Ganz, 2004). Experimental evidence is needed to support these observational data and clarify the mechanisms by which gonadotropins contribute to endothelial dysfunction in women across the adult lifespan.

#### 3.2.4 Testosterone

Endogenous total testosterone and SHBG are not correlated with FMD in women of all menopausal stages (age 22–75) (Moreau et al., 2012). Interestingly, free testosterone is inversely associated with FMD, and SHBG is positively associated with FMD, in peri‐ and postmenopausal women (Mathews et al., 2019). This is consistent with a prospective cohort study in postmenopausal women demonstrating that higher free androgen index (testosterone:SHBG) was associated with lower FMD after ∼1–2 years’ follow‐up (Georgiopoulos et al., 2016). Exogenous testosterone administration improved FMD in postmenopausal women already on HT (Worboys et al., 2001), suggesting that concomitant oestradiol administration may modulate the effect of testosterone on endothelial function in postmenopausal women. As with oestradiol, testosterone receptors are expressed in endothelial cells and regulate endothelial function through both transcriptional and non‐transcriptional pathways that augment NO production (Yu et al., 2012). It is important to note that these studies were conducted in male tissue lines, and there is significant sexual dimorphism in endothelial cell phenotypes (Shin et al., 2024). Future study is needed to elucidate the complex interactions between declines in oestradiol and altered androgen bioavailability with regard to vascular function in menopausal women.

### 3.3 Integrative mechanisms by which sex hormones modulate endothelial function

#### 3.3.1 Endothelin‐1 and endothelin receptor function

ET‐1 is produced by the endothelium and acts through autocrine and paracrine signalling in the vasculature via ET type A receptors (ET_A_R) and ET type B receptors (ET_B_R). Both receptors are present on vascular smooth muscle cells and cause vasoconstriction; ET_B_R are also expressed on the vascular endothelium and induce vasodilation. Accordingly, the effect of ET‐1 on vascular tone is the net result of vascular smooth muscle ET_A_R and ET_B_R‐mediated vasoconstriction and endothelial ET_B_R‐mediated vasodilation (Thorin & Clozel, 2010).

Oestradiol modulates ET‐1 system signalling via both ET‐1 production and receptor function. Oestradiol attenuates ET‐1 synthesis release in human endothelial cells (Bilsel et al., 2000), and promotes ET_B_R‐mediated vasodilation. Indeed, perfusion of an ET_B_R antagonist blunts endothelium‐dependent cutaneous microvascular vasodilation during the midluteal (i.e., elevated endogenous oestradiol and progesterone levels) but not during the early follicular phase (oestradiol and progesterone nadir) in natural cycling premenopausal women (Sebzda et al., 2018). Endothelium‐dependent cutaneous microvascular vasodilation in response to ET_B_R blockade is similarly attenuated by exogenous oestradiol administration in premenopausal women during a controlled hormone intervention (i.e., endogenous hormone suppression) (Shoemaker et al., 2021). Collectively, these studies in premenopausal women indicate that oestradiol promotes ET_B_R‐mediated vasodilation.

**FIGURE 4:**
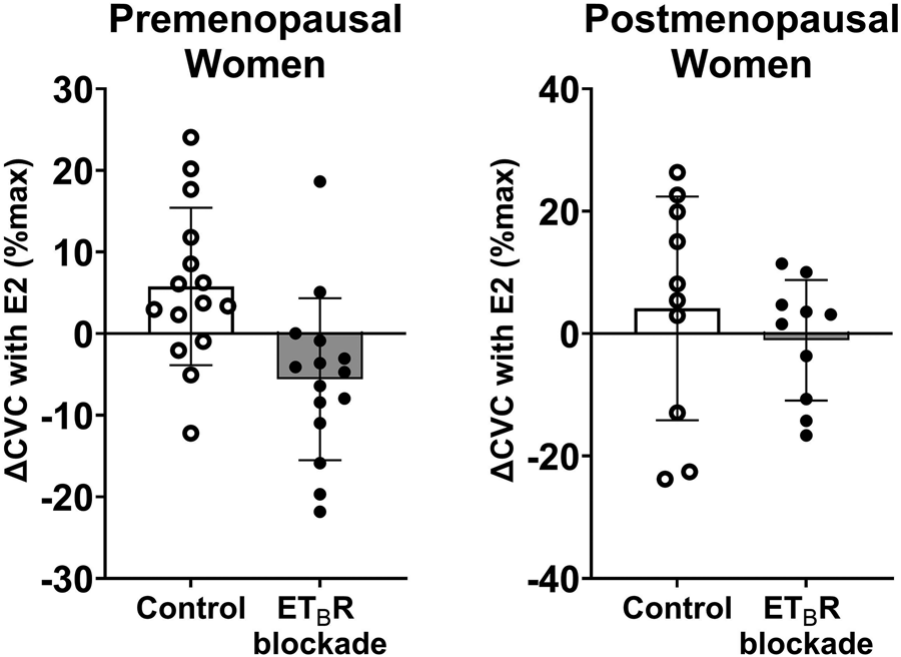
Oestradiol modulates endothelin‐R receptor (ET_B_R)‐mediated vasodilation in premenopausal women, but oestradiol does not restore ET_B_R‐mediated vasodilation in postmenopausal women. Cutaneous vascular conductance was measured before and after 7 days of exogenous oestradiol (E2) administration (0.1 mg/day) among premenopausal women undergoing suppression of endogenous oestradiol production via gonadotropin‐releasing hormone antagonist (Shoemaker et al., 2021) and oestrogen‐deficient postmenopausal women (Nuckols et al., 2025). The figure shows change in the cutaneous microvascular vasodilatory response (ΔCVC) to local heating alone (white bars) or with perfusion of ET_B_R antagonist BQ‐788 (grey bars) elicited by exogenous E2 during the controlled hormone intervention.

Declines in oestradiol and ET‐1 receptor function contribute to impaired endothelial function observed in postmenopausal women. ET‐1 is higher in post‐ compared with premenopausal women, and ET_B_R‐mediated vasodilation is lost in women after menopause (Wenner et al., 2017). This loss of ET_B_R‐mediated endothelial function is likely driven by lower endothelial ET_B_R expression in post‐ compared with premenopausal women (Kuczmarski et al., 2020). Interestingly, exogenous oestradiol administration does not restore ET_B_R‐mediated vasodilation in postmenopausal women (Nuckols et al., 2025), in contrast to observations in premenopausal women (Figure 4). This finding may be explained in part by emerging preclinical evidence that GPER regulates renal ET‐1 expression in female rodents (Guthrie et al., 2023), suggesting crosstalk between oestradiol and ET‐1 receptor systems. Further translational research is needed to understand the mechanisms that explain the loss of the cardioprotective effect of oestradiol in ET‐1 system signalling after menopause.

The physiological relevance of other sex hormones to ET‐1 production and receptor function in postmenopausal women has not been well established. In human endothelial cells, progesterone treatment reduces ET‐1 secretion and withdrawal promotes ET‐1 release, suggesting that progesterone may have similar effects to oestradiol on ET‐1 production (Edlund et al., 2004). It is important to note that like oestradiol (Gohar et al., 2016), the effect of progesterone on ET‐1 signalling may be tissue specific (Edlund et al., 2004) and to date has been characterized primarily in reproductive vascular beds. Data from our labs demonstrate a direct bivariate relationship between FSH and plasma ET‐1 in women over the lifespan (*R* ^2^ = 0.06), suggesting functional implications of FSH for ET‐1‐mediated endothelial function in women (Wenner & Moreau, unpublished data). Women with polyendocrine metabolic ovarian syndrome (previously named polycystic ovary syndrome, characterized by excess testosterone) demonstrate attenuated ET_B_R‐mediated dilation compared with control women (Wenner et al., 2011).

#### 3.3.2 Oxidative stress and inflammation

Key mechanisms of endothelial dysfunction in populations of varied health status (i.e., primary ageing, hypertension) are oxidative stress and inflammation. For comprehensive review of this literature, the reader is referred to Dinh et al. (2014). Briefly, oxidative stress is characterized by imbalanced reactive oxygen species (ROS) relative to the capacity of antioxidants to detoxify ROS. Accumulation of ROS contributes to endothelial dysfunction via inhibition of eNOS and scavenging of NO. ROS are involved in the inflammatory cascade. Inflammatory cytokines (e.g., tumour necrosis factor‐α, TNF‐α) suppress eNOS activity and contribute to production of ROS. Interaction between these pro‐oxidative, pro‐inflammatory pathways results in an overall reduction in NO bioavailability and endothelial dysfunction (Dinh et al., 2014).

Biomarkers of oxidative stress and systemic inflammation are elevated in postmenopausal compared with premenopausal women independent of chronological age and traditional CVD risk factors (Heravi et al., 2022). Indeed, in peri‐ and postmenopausal women, oestradiol is inversely related to markers of oxidative stress and positively correlated with antioxidant enzyme levels (Ogunro et al., 2014). Experimental evidence demonstrates that infusion of the antioxidant ascorbic acid (vitamin C), an approach that acutely reduces ROS, improves endothelial function in late perimenopausal and postmenopausal women but has no effect in pre‐ and early perimenopausal women (DuBose et al., 2022; Moreau et al., 2020). Further, vitamin C infusion restored the impaired endothelial function induced by pharmacological ovarian hormone suppression in pre‐ and perimenopausal women but did not alter FMD during the exogenous oestradiol administration condition (Figure 3). Similarly, systemic administration of a TNF‐α inhibitor (i.e., anti‐inflammatory agent) improved FMD in oestradiol‐deficient postmenopausal women, but not in premenopausal women (Moreau et al., 2013). These studies consistently demonstrate that antioxidant and anti‐inflammatory agents improve FMD selectively in oestradiol‐deficient postmenopausal women. Accordingly, increased oxidative stress and inflammation attributable in part to the loss of oestradiol is likely causally linked to impaired endothelial function with the menopause transition.

The mechanisms by which oestradiol mitigates the effect of oxidative stress and inflammation are multifaceted. As reviewed above, oestradiol promotes NO production, which has both antioxidant and anti‐inflammatory processes (Moncada & Higgs, 2006). Oestradiol has direct antioxidant properties enabling scavenging of ROS (Hernández‐Hernández et al., 2023). Additionally, oestradiol upregulates the expression and activity of antioxidant enzymes and suppresses the expression of pro‐oxidant enzymes (Strehlow et al., 2003). Oestradiol exposure downregulates expression of inflammatory biomarkers (e.g., TNF‐α, interleukin (IL)‐1β, IL‐6, IL‐8) in uterine artery segments from postmenopausal women. Interestingly, this anti‐inflammatory effect of oestradiol was limited to early postmenopausal women for select inflammatory markers (e.g., IL‐6, IL‐8). In contrast, oestradiol exposure demonstrated an ERβ‐mediated pro‐inflammatory effect in late postmenopausal women. Female endothelial cells exhibit higher basal ROS levels and expression of genes associated with inflammation compared with male endothelial cells (Shin et al., 2024). Accordingly, menopause‐related loss of oestradiol may ‘unmask’ a vulnerable pro‐oxidant, pro‐inflammatory phenotype and shifts in oestradiol receptor profiles may contribute to the deleterious effect of oestradiol observed in later menopause stages.

The direct, independent impacts of other sex hormones including progesterone, FSH, LH and testosterone on oxidative stress or inflammation in postmenopausal women have not been widely studied. Similar to oestradiol, progesterone is associated with lower markers of oxidative stress and greater antioxidant enzyme activity in postmenopausal women (mean age 57) (Ogunro et al., 2014). FSH and LH exhibited the opposite pattern, wherein both hormones were correlated with biomarkers of oxidative stress and lower antioxidant enzyme activity. These patterns suggest that in conjunction with declines in oestradiol, declines in progesterone and increases in FSH and LH during the menopause transition may promote oxidative stress. Interestingly, the correlations between FSH and LH with both pro‐ and antioxidant markers were stronger than those of oestradiol and progesterone. In the Postmenopausal Estrogen/Progestin Interventions (PEPI) trial cohort, progesterone and free testosterone levels were cross‐sectionally related with select markers of inflammation in postmenopausal women, but associations were not present across all markers (i.e., C‐reactive protein, IL‐6, matrix metalloproteinase, and soluble intercellular adhesion molecule) (Crandall et al., 2006). These inconsistent correlational data limit causal inference, and the independent effects of these hormones are indeterminable. However, preclinical models support the direct role of progesterone and FSH in oxidative stress and inflammatory processes, respectively, in select non‐vascular tissues. In ovariectomized rodents, a preclinical model of menopause, progesterone administration prevented the pro‐oxidant effect of chronic intermittent hypoxia, suggesting an independent antioxidant effect of progesterone (Joseph et al., 2020). FSH upregulated the expression of proinflammatory cytokines in in vitro connective and immune cells lines (Cannon et al., 2010; Qian et al., 2020). Moreover, ovariectomized rodents exhibited augmented proinflammatory cytokine secretion compared with sham rodents, whereas this effect was prevented with administration of an FSH inhibitor (Qian et al., 2020). Taken together, convergent in vitro studies, rodent models of menopause, and observational findings in postmenopausal women provide nascent evidence for the role of sex hormones other than oestradiol in driving oxidative stress and inflammatory processes with menopause. However, the independent effects of individual sex hormones have not been characterized in postmenopausal women, and the downstream implication for the endothelial function is not known.

## 4 SUMMARY AND FUTURE DIRECTIONS

The appreciable reduction in vascular endothelial function observed during the menopause transition corresponds with declines in oestradiol and progesterone concurrent with elevations in FSH and LH. The protective effects of oestradiol on endothelial function are well‐established and mediated via integrative mechanisms including ET‐1 receptor signalling, oxidative stress and inflammatory pathways. The independent contributions of other sex hormones including progesterone, FSH, LH and testosterone to vascular ageing with menopause, and the underlying mechanistic pathways, remain insufficiently characterized. Evidence from both observational associations with endogenous hormones and exogenous hormone administration studies supports the vascular effects of oestradiol, and to a lesser extent other sex hormones, but the distinct effects of varied formulations require further investigation. Moreover, it is important to note that the majority of studies have quantified endogenous serum hormone concentrations via widely used immunoassay‐based methods, which are less sensitive than mass spectrometry methods at the low hormone concentrations characteristic after menopause. This represents an important emerging area for future study, as novel evidence suggests that these sex hormones are involved in endothelial dysfunction to a comparable or greater extent than oestradiol via similar pathways. Future research is needed to understand how sex hormones beyond oestradiol modulate vascular health across the lifespan in women, such as studies to (1) isolate the effects of individual sex hormones on endothelial function in menopausal women through controlled hormone interventions and precise quantification techniques; (2) disentangle the signalling pathways by which sex hormones, individually or by interaction, exert their effects on the vasculature; and (3) investigate clinical applications including optimal timing for hormonal intervention. Addressing these knowledge gaps is needed to advance therapeutic approaches for CVD prevention in menopausal women.

## AUTHOR CONTRIBUTIONS

Conceptualization (Virginia R. Nuckols and Megan M. Wenner); Visualization (Virginia R. Nuckols and Allyson I. Schwab); Writing—original draft (Virginia R. Nuckols and Megan M. Wenner); Writing—review & editing (Virginia R. Nuckols, Allyson I. Schwab., Lyndsey E. DuBose, Kerrie L. Moreau, and Megan M. Wenner). All authors have read and approved the final version of this manuscript and agree to be accountable for all aspects of the work in ensuring that questions related to the accuracy or integrity of any part of the work are appropriately investigated and resolved. All persons designated as authors qualify for authorship, and all those who qualify for authorship are listed.

## CONFLICT OF INTEREST

None declared.
